# Predictive Ability for Drug Use in Sub-Clinical Students with Attention Deficit Hyperactivity Disorder by Questionnaire Survey

**DOI:** 10.5812/ijhrba.9578

**Published:** 2013-06-26

**Authors:** Tomoyuki Kawada

**Affiliations:** 1 Department of Hygiene and Public Health, Nippon Medical School, Tokyo, Japan

**Keywords:** Attention Deficit Disorder with Hyperactivity, Alcoholism, Addiction

Dear Editor,

I read with interest the article by Bayrami et al., reporting the predictive ability of drug use for subjects with attention deficit hyperactivity disorder (ADHD) symptoms (1). The target 361 subjects were sampled randomly from 5129 university students, and a cross-sectional survey was conducted by using a questionnaire. Recently, Lichtenstein et al. conducted a follow-up study to know the effect of medication for 25,656 ADHD patients on criminality (2). They concluded that the use of medication for ADHD reduces significantly the risk criminality. These two surveys contain completely different population from the viewpoint on the severity of ADHD. Although Bayrami et al. mentioned that there was a positive relationship between ADHD symptoms and drug use, a further study is needed to make a finalconclusion. First, their study is a cross-sectional study, and cause-effect relationship cannot be defined. Second, the target subjects are composed mainly of healthy young population, and clinical diagnosis of ADHD has not been made. Third, Pearson's moment correlation coefficient using scores from the questionnaire on ADHD and addiction acknowledgment scale score or Mac Andrew alcoholism scale scores showed under 0.4. This means that the relationship is not so strong, although a significant relationship was observed. This fact was also clarified by the multiple regression analysis, which was presented in Tables 2 and 3. Adjusted square values of R (multiple correlation coefficients) were under 0.16, which meant that each explanation rate was under one-sixthof all the variance. Before describing the predictive ability as they conducted, I recommend to describe the characteristics of the target university students. By this process, trace ability of their results by other researchers would improve. In anyway, there is a need for more studies to conclude the effect of ADHD symptoms on drug use including alcoholism. 
